# Small fiber neuropathy in Parkinson's disease: A clinical, pathological and corneal confocal microscopy study

**DOI:** 10.1016/j.parkreldis.2015.10.019

**Published:** 2015-12

**Authors:** Lewis Kass-Iliyya, Saad Javed, David Gosal, Christopher Kobylecki, Andrew Marshall, Ioannis N. Petropoulos, Georgios Ponirakis, Mitra Tavakoli, Maryam Ferdousi, Kallol Ray Chaudhuri, Maria Jeziorska, Rayaz A. Malik, Monty A. Silverdale

**Affiliations:** a Department of Neurology, Greater Manchester Neuroscience Centre, Salford Royal NHS Foundation Trust, Salford, UK; b Institute of Brain, Behaviour and Mental Health, University of Manchester, Manchester, UK; c Institute of Human Development, University of Manchester, Manchester, UK; d Weill Cornell Medical College, Doha, Qatar; e Department of Clinical Neuroscience, King's College, London, UK

**Keywords:** Parkinson's disease, Cornea, Confocal microscopy, Small fiber neuropathy, Intraepidermal nerve fiber density

## Abstract

Autonomic and somatic denervation is well established in Parkinson's disease (PD).

**Objectives:**

(1) To determine whether corneal confocal microscopy (CCM) can non-invasively demonstrate small nerve fiber damage in PD. (2) To identify relationships between corneal nerve parameters, intraepidermal nerve fiber density (IENFD) and clinical features of PD.

**Methods:**

Twenty-six PD patients and 26 controls underwent CCM of both eyes. 24/26 PD patients and 10/26 controls underwent skin biopsies from the dorsa of both feet. PD patients underwent assessment of parasympathetic function [deep breathing heart rate variability (DB-HRV)], autonomic symptoms [scale for outcomes in Parkinson's disease – autonomic symptoms (SCOPA-AUT)], motor symptoms [UPDRS-III “ON”] and cumulative Levodopa dose.

**Results:**

PD patients had significantly reduced corneal nerve fiber density (CNFD) with increased corneal nerve branch density (CNBD) and corneal nerve fiber length (CNFL) compared to controls. CNBD and CNFL but not CNFD correlated inversely with UPDRS-III and SCOPA-AUT. All CCM parameters correlated strongly with DB-HRV. There was no correlation between CCM parameters and disease duration, cumulative Levodopa dose or pain. IENFD was significantly reduced in PD compared to controls and correlated with CNFD and UPDRS-III. However, unlike CCM measures, IENFD correlated with disease duration and cumulative Levodopa dose but not with autonomic dysfunction.

**Conclusion:**

CCM identifies corneal nerve fiber pathology, which correlates with autonomic symptoms, parasympathetic deficits and motor scores in patients with PD. IENFD is also reduced and correlates with CNFD and motor symptoms but not parasympathetic deficits, indicating it detects different aspects of peripheral nerve pathology in PD.

## Highlights

• Peripheral denervation is established in Parkinson's disease (PD) on skin biopsies.
• Corneal confocal microscopy (CCM) can non-invasively visualize corneal nerves.
• We have shown significant corneal denervation in PD compared to controls using CCM.
• Corneal denervation relates to skin denervation and parasympathetic dysfunction.

## 1 Introduction

Parkinson's disease (PD) is a neurodegenerative disease recognized clinically by its motor symptoms of rest tremor, bradykinesia, rigidity and postural instability. The motor syndrome results from degeneration of dopaminergic neurons in the substantia nigra. Abnormal aggregates of alpha-synuclein in neuronal cells form the histopathological hallmark of PD.

A substantial body of evidence demonstrating peripheral nerve pathology has challenged the traditional view of PD as a disorder of the central nervous system. Phosphorylated alpha-synuclein deposits have been demonstrated in the autonomic nerves of the colon, cardiac plexus and more recently cutaneous C fibers [1], [2], [3], [4], [5].

Several studies have shown clinical and histopathological evidence of small and large fiber peripheral neuropathy in PD [6], [7], [8], [9], [10]. To date there is no consensus on the underlying pathophysiology of peripheral neuropathy in PD. Cumulative Levodopa exposure has been proposed as a potential cause for large fiber neuropathy via homocysteine accumulation and reduced levels of folate as well as vitamin B12 [6], [9], [10]. However several studies have found no relation between peripheral denervation and Levodopa exposure suggesting that peripheral nerve involvement may be an intrinsic feature of the disease, especially in relation to small fiber neuropathy [1], [7], [11]. Furthermore, the identification of alpha-synuclein in cutaneous nerves has led to the consideration of skin tissue as a potential biomarker in PD [2], [5].

The cornea receives its innervation from the ophthalmic branch of the trigeminal nerve. Corneal nerves have an important neurotrophic role in maintaining corneal integrity. Small unmyelinated nerve fibers enter the cornea from the periphery and course through the corneal stroma before they penetrate the sub-epithelial Bowman's membrane, giving rise to the sub-basal nerve plexus [12]. This nerve plexus is of particular relevance for defining neuropathic changes since it supplies the overlying corneal epithelium. We have developed corneal confocal microscopy (CCM) as a rapid, non-invasive technique for *in vivo* visualization of corneal nerves and shown it to be a valid surrogate measure of diabetic somatic and autonomic neuropathy [13], [14]. CCM can also detect early neuropathy in patients with recently diagnosed type 2 diabetes and subjects with impaired glucose tolerance [15], [16]. Furthermore, CCM has a high sensitivity in detecting early nerve regeneration following simultaneous pancreas and kidney transplantation in diabetic neuropathy [17]. We have further extended the role of CCM to detect small fiber neuropathy in Fabry's disease, idiopathic small fiber neuropathy and Charcot–Marie–Tooth 1A [18], [19], [20].

One study that included 4 patients with PD reported no significant reduction in corneal nerve density compared to controls [21]. However, assessment of corneal nerve morphology was not the primary aim of the study and the small number of PD participants precludes meaningful conclusions regarding the utility of CCM in PD.

Given the strong evidence for small fiber neuropathy in PD, we hypothesized that CCM would allow rapid, non-invasive detection of corneal nerve pathology and that this would be related to intraepidermal nerve fiber density and clinical symptoms in PD, in particular those related to pain and autonomic dysfunction.

## 2 Methods

### 2.1 Ethical approval

NRES Committee/North West (Ref. No 12/NW/0086) approved the study.

### 2.2 Subjects

Thirty-three patients (22 males, 11 females) fulfilling the UK Brain Bank criteria for the diagnosis of Parkinson's disease were recruited from neurology clinics. Patients with a known history of cancer, chemotherapy, diabetes, alcoholism, vitamin deficiencies, celiac disease and autoimmune conditions were excluded. All patients underwent an oral glucose tolerance test and fasting blood screening to include: vitamin B12, folate, methylmalonate, and homocysteine to exclude other causes of neuropathy. Six patients (5 males, 1 female) were excluded due to impaired glucose tolerance, leaving 27 patients who fulfilled the inclusion criteria. CCM was not undertaken in 1 patient due to previous refractive eye surgery. Twenty-six age-matched healthy volunteers served as controls. All participants gave their written informed consent.

### 2.3 Disease duration, severity and levodopa exposure

PD duration was calculated from reported symptoms onset until the date of assessment. UPDRS-III was used to assess motor severity. Patients were examined in the “ON” state in an outpatient setting. All patients except one were on Levodopa therapy. Cumulative Levodopa dose was calculated from the date of first prescription until the date of assessment.

### 2.4 Non-motor symptoms

Non-motor symptoms were quantified using the non-motor symptoms scale NMSS [22]. Pain was quantified separately using the recently devised King's PD pain scale [23] as well as the short form McGill Pain Questionnaire (SFMPQ).

### 2.5 Autonomic symptoms and function

Autonomic symptoms were assessed using the scale for outcomes in Parkinson's disease – autonomic symptoms (SCOPA-AUT) [24]. Deep breathing heart rate variability (DB-HRV) provided an estimation of cardiac parasympathetic dysfunction and was assessed with an ANX 3.0 autonomic nervous system devise (ANSAR Medical Technologies Inc., Philadelphia, PA). Cardiovascular sympathetic function was assessed by measuring blood pressure and heart rates in the supine position and after standing up for 5 min.

### 2.6 Evaluation of neuropathy

Nerve conduction studies were performed on all PD patients. Patients with evidence of a large fiber neuropathy underwent assessment of immunoglobulins and protein electrophoresis to rule out the presence of a paraproteinemic neuropathy. The neuropathy disability score (NDS) was obtained in all participants. NDS is a bedside measure to stratify diabetic neuropathy [25]. A clinician assesses vibration perception using a tuning fork, temperature perception using a cold and warm metal rods, pinprick perception using Neurotips™ and ankle reflexes using a tendon hammer producing a score from 0 to 10. Quantitative Sensory Testing was assessed using the MEDOC TSA II (Medoc Ltd., Ramat-Yishai 20095, Israel) device on the dorsum of the left foot. Quantitative sensory testing included heat sensation threshold, heat-induced pain threshold, cold threshold and cold-induced pain threshold. Vibration perception threshold was measured using a Neurothesiometer (Horwell, Scientific Laboratory Supplies, Nottingham, UK) on the hallux of both feet.

### 2.7 Skin biopsies

Two 3-mm punch skin biopsies were taken from the dorsa of both feet. The biopsies were immediately fixed in 4% paraformaldehyde, cryoprotected in graded solutions of sucrose, frozen and cut on a cryomicrotome (HM450, Microm International, Germany). Six 50 μm sections per biopsy were immuno-stained using anti-human PGP 9.5 antibody and nerve fibers were demonstrated using SG chromogen (Vector Laboratories, Peterborough, U.K.). A pathologist blinded to participants' details performed tissue analysis. Intraepidermal nerve fiber density (IENFD), i.e., the number of nerve fibers crossing basement membrane, was quantified according to established criteria and expressed as number per millimetre of epidermal length [26]. The mean between right and left IENFD was calculated for each patient and used for analysis.

### 2.8 Corneal confocal microscopy

Corneal microscopy was performed on both eyes using a Heidelberg Retina Tomograph III with a Rostock Cornea Module (HRT III RCM; Heidelberg Engineering GmbH, Heidelberg, Germany), as previously described [17]. Four to six high-resolution (1–2 μm) images of the sub-basal plexus of each eye were obtained for all participants. A trained investigator who was blinded to participants' details analysed corneal images separately. Corneal Nerve Fiber Density (CNFD): The number of main nerves per square millimetre, Corneal Nerve Branch Density (CNBD): The number of branches emanating from each main nerve per square millimetre and Corneal Nerve Fiber Length (CNFL): The length of all nerve fibers and branches (mm per square millimetre) were quantified and the mean derived from the right and left eye for each parameter. Quantification was undertaken using semi-automated, purpose-written, proprietary software (CCMetrics; M.A. Dabbah, Imaging Science and Biomedical Engineering, Manchester, UK). To estimate the error in measuring CNFD, CNBD and CNFL, we acquired images and determined each of these parameters in 15 subjects on two occasions separated by at least 48 h. The coefficient of variation of these parameters was 12% for CNFD, 24% for CNBD and 9% for CNFL.

### 2.9 Statistical analysis

IBM SPSS version 22 was used to compute the results. All parameters were normally distributed except for cumulative Levodopa dose, vibration and cold perception thresholds as well as heat and cold pain thresholds. Independent samples *t*-test or Mann–Whitney U test were used to compare means as appropriate. Cohen *d* was calculated to measure effect size. Two-tailed Pearson's correlation or Spearman's correlations were used as appropriate to determine relationships between continuous variables. Categorical data were compared with Chi square test. *P* value of <0.05 is considered statistically significant.

## 3 Results

### 3.1 Study population

A total of 27 PD patients and 26 controls were enrolled in the study. There was no significant difference in demographics between PD patients and control subjects (Table 1).

### 3.2 Corneal and intraepidermal nerve fibers

**Fig. 1:**
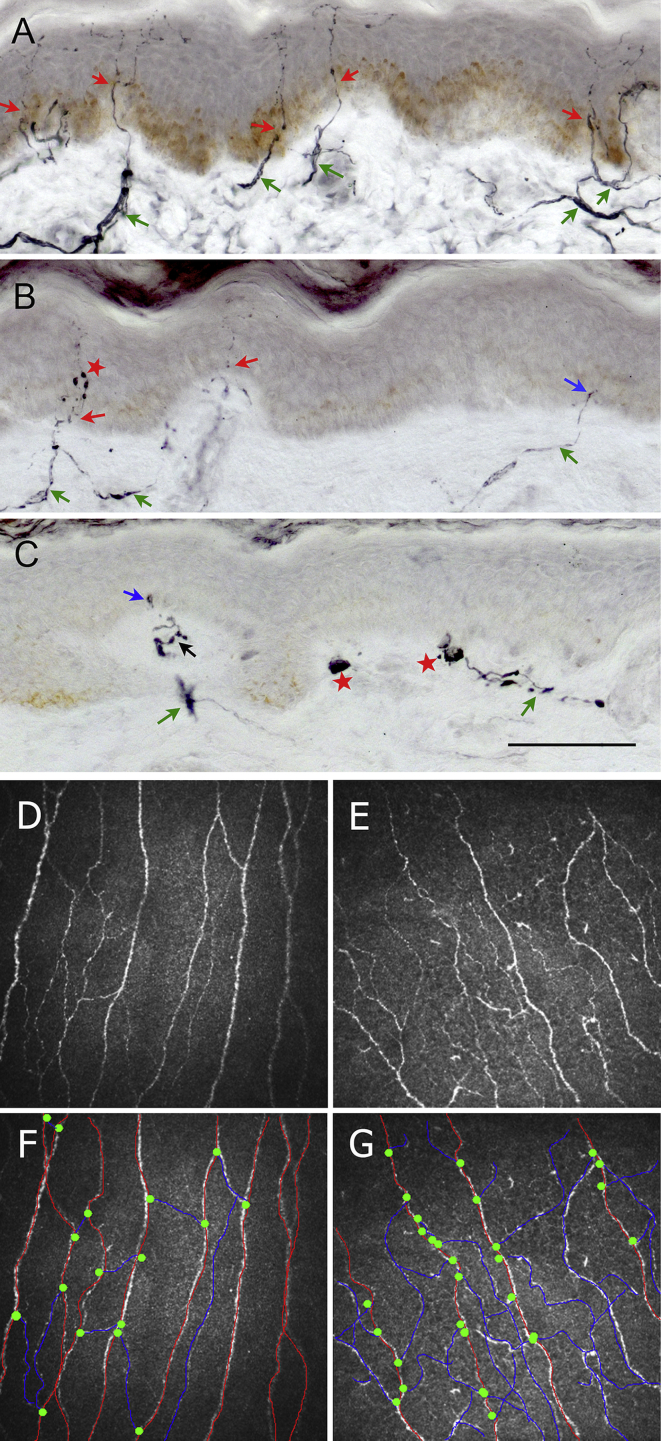
Representative examples of 50 μm sections from skin biopsies immunostained for PGP9.5. Healthy control (A) shows numerous long branching intraepidermal nerve fibers (red arrows) reaching upper layers of epidermis and well developed sub-epidermal nerve plexus (green arrows). Biopsy from a patient with Parkinson's disease (B) showing scant, faintly staining intraepidermal nerve fibers (red arrows), some branches with axonal swellings (red star) and a biopsy from another patient with Parkinson's disease (C) showing an area of epidermis with only one short intraepidermal nerve fiber and signs of degeneration in the sub-epidermal nerve plexus (red stars). Note short nerve fibers, barely crossing epidermal basement membrane (B, C, blue arrows). A–C at the same magnification, scale bar = 100 μm. Corneal confocal image of a healthy control (D) compared to a patient with Parkinson's disease (E) showing a reduction in overall corneal nerve fiber density and increased corneal nerve branch density. Quantification of corneal nerve fiber density, branch density and fiber length is calculated using a semi-automated process. Main fibers are highlighted in red, branches are highlighted in blue and branch origins are represented by the green dots (F&G). (For interpretation of the references to colour in this figure legend, the reader is referred to the web version of this article.)

**Fig. 2:**
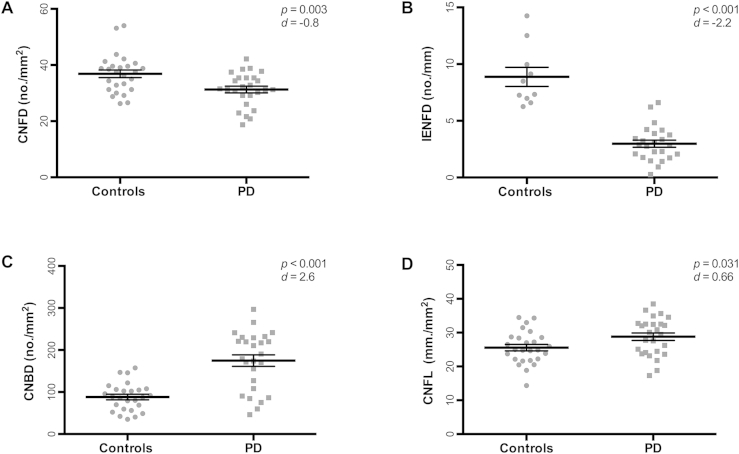
Mean ± SEM of corneal nerve fiber density (CNFD) (A), intraepidermal nerve fiber density (IENFD) (B), corneal nerve fiber branch density (CNBD) (C) and fiber length (CNFL) (D) in Parkinson's disease (PD) compared to controls with significance level and effect size.

Twenty-six PD patients and 26 controls underwent CCM and 24 PD patients and 10 controls underwent skin biopsies. Both CNFD and IENFD were significantly lower in PD patients compared to controls (CNFD mean difference: −5.6 no./mm^2^, 95% CI [−9.2, −2], *P* = 0.003; IENFD mean difference: −5.9 no./mm, 95% CI [−7.9, −3.9], *P* < 0.001). However, CNBD was significantly higher in PD patients compared to controls (CNBD mean difference: 86.6 no./mm^2^, 95% CI [55.9, 117.2]), *P* < 0.001). CNFL was also significantly higher in PD patients (CNFL mean difference: 3.2 mm./mm^2^, 95% CI [0.3, 6.1], *P* = 0.031) (Fig. 1, Fig. 2).

Both IENFD and CCM parameters displayed asymmetry between sides in PD patients. However, when CCM measures and IENFD were separated according to the clinically more affected side (defined by UPDRS-III scores) and the clinically less affected side there was no significant difference in CCM measures or IENFD.

### 3.3 Clinical, neurophysiological, quantitative sensory and autonomic measures of neuropathy

NDS, DB-HRV and sensory thresholds all showed impairment in PD compared to controls (Table 1). Overall, there was no significant postural drop in blood pressure in PD (defined as mean systolic drop of >20 mm Hg and/or diastolic drop of >10 mm Hg) (Table 1). Three out of 27 PD patients (11.1%) had large fiber axonal neuropathy on nerve conduction studies and 2 of these patients had raised methylmalonate. However, methylmalonate was raised in a further 4 PD patients with normal nerve conduction studies. Homocysteine was raised in 5 (18.5%) patients all of whom had normal neurophysiology. All patients had normal vitamin B12 and folate levels. Protein electrophoresis was normal in patients with large fiber neuropathy.

### 3.4 Relation between corneal nerve parameters, intraepidermal nerve fibers and clinical features

CNFD correlated positively with IENFD in PD patients (Pearson's *r* = 0.464, *P* = 0.026). Correlations between CCM parameters, IENFD and clinical data are summarized in Table 2. CNBD and CNFL but not CNFD correlated inversely with UPDRS-III. There was no correlation between any of the corneal nerves parameters and disease duration, cumulative Levodopa dose, non-motor symptoms, pain scales or sensory thresholds. However, there was an inverse correlation between heat-induced pain threshold and SFMPQ (Spearman's rho −0.450, *P* = 0.036). A significant positive correlation was found between DB-HRV and all corneal nerve parameters, but not IENFD (Table 2). However, IENFD correlated with disease duration, disease severity and cumulative Levodopa dose. Both CNFD and IENFD correlated with NDS. CNFD, IENFD and NDS were independent of vitamin B12, folate, methylmalonate and homocysteine levels.

## 4 Discussion

This is the first study to systematically characterize corneal nerve changes in PD using the non-invasive technique of corneal confocal microscopy. Compared to controls, PD patients demonstrated a reduction in CNFD, a marked increase in CNBD and an increase in CNFL. This pattern of corneal nerve pathology is notably different from diabetes, Fabry's disease, chronic inflammatory demyelinating polyneuropathy and Charcot–Marie Tooth 1A, where a reduction of all three corneal nerve parameters has been documented [13], [18], [20], [27]. Thus the reduced CNFD with a markedly increased CNBD appears to be unique in PD. Pathologically this represents small fiber neuropathy, characterized by the reduced CNFD. However, the increased CNBD is likely to reflect attempted nerve regeneration. Increased branching of nerve fibers in PD has been previously reported on skin biopsies signifying attempts at nerve regeneration [8]. In our PD patients CNBD correlated inversely with motor severity suggesting that nerve regeneration occurs in the early stages of the disease and declines as the disease becomes more advanced. Indeed, increased corneal nerve branch density was the first measure to increase after simultaneous pancreas and kidney transplantation or continuous subcutaneous insulin infusion in type 1 diabetes [17], [28].

We confirm the results of a number of studies, which have shown a reduction of distal IENFD in PD patients [5], [7], [8]. However, we are the first to report a reduction of CNFD, which correlates with IENFD indicating widespread involvement of small nerve fibers in PD. This is further supported by the significant impairment of modalities relating to both parasympathetic and sensory functions (DB-HRV, thermal perception and pain thresholds). We did not document significant postural drop in blood pressure or heart rate (sympathetic cardiovascular function) in our PD population. Given that postural hypotension is a manifestation of severe sympathetic dysfunction this may reflect the disease stage of most of our PD cohort with relatively few patients in very advanced stages (Table 1).

The change in corneal nerve parameters was independent of age, cumulative Levodopa dose, methylmalonate, homocysteine, B12 and folate levels, suggesting that damage to corneal nerves is a result of an intrinsic disease process rather than external factors. Furthermore, unlike diabetes where corneal nerve damage was symmetrical [29] we found asymmetry in corneal nerve parameters between sides in PD patients suggesting that corneal neuropathy in diabetes is due to a global metabolic process, whereas in PD may be due to a more localized degenerative process consistent with PD pathology.

Corneal nerve changes strongly correlated with autonomic symptoms (SCOPA-AUT) and parasympathetic dysfunction (DB-HRV), but did not correlate with disease duration or cumulative Levodopa dose. However, IENFD correlated with disease duration, and cumulative Levodopa dose but not with autonomic symptoms or parasympathetic deficits (Table 2). These findings suggest that CCM and skin biopsies may be reflecting different aspects of neuronal pathology in PD and indeed corneal nerves changes rather than IENFD are more closely associated with autonomic pathology. In support of this we have recently shown that CCM has a very high sensitivity and specificity for the diagnosis of diabetic autonomic neuropathy and correlates with autonomic symptoms and deficits [14]. The moderate correlation between IENFD and CNFD emphasizes that skin biopsy and CCM are visualizing different aspect of small fiber pathology. Indeed in a recent study of 134 patients with early diabetic neuropathy the association between IENFD and CCM pathology was even lower [16]. It would be interesting to investigate whether corneal denervation is present during the pre-motor phase when autonomic symptoms are commonly reported which can potentially add to the sensitivity and specificity of pre-motor features such as anosmia and REM sleep behaviour disorder in diagnosing pre-motor PD.

Previous studies of skin biopsies in PD have revealed preferential deposition of phosphorylated alpha-synuclein in autonomic cutaneous nerves, with a higher yield for alpha-synuclein from proximal as opposed to distal sites [2], [5]. IENFD reduction, on the other hand, follows a length-dependent pattern [7]. This has led to the suggestion that alpha-synuclein deposition and a length-dependent reduction in IENFD are different pathological processes [7]. Distal skin denervation in our PD patients was more pronounced than corneal denervation (Fig. 2). Taken together one may conclude that the difference of alpha-synuclein deposition between proximal and distal sites may reflect neuronal availability with distal axons being more vulnerable to degeneration compared to proximal sites.

Similar to previous reports we found otherwise unexplained clinical and neurophysiological evidence of large fiber neuropathy in a proportion of our PD patients but we could not establish a link between levodopa exposure or vitamin deficiencies and large fiber neuropathy. The question of Levodopa induced neuropathy in PD remains a subject of debate. While there have been reports linking large fiber neuropathy with prolonged exposure to Levodopa [6], [9], evidence for such a link with small fiber neuropathy is lacking [1], [2], [7], [11]. Although we have found a correlation between IENFD and cumulative Levodopa dose, establishing causality is difficult as reduced IENFD also correlated with disease duration and severity. Correcting for these factors using multiple regression analysis may address this question, but our study was not powered for this. However, given the lack of relationship between changes in IENFD and methylmalonate, homocysteine or B12 as well as the asymmetry between skin biopsies, our results suggest that cutaneous denervation may be due to an intrinsic disease process rather than an extrinsic toxicity due to Levodopa.

Pain is very common in PD, affecting up to 70% of PD patients [30]. We found no correlation between self-reported pain scores and small fiber nerve density suggesting that small fiber pathology may make a lower contribution to pain in PD and that more central mechanisms are involved. This is further supported by the inverse correlation between heat-induced pain threshold and SFMPQ indicating a reduced pain threshold in painful PD phenotypes. Thus pain in PD is more likely to be due to an impaired central processing of nociceptive inputs rather than damaged peripheral nerves.

Both CNBD and CNFL as well as IENFD correlated negatively with UPDRS-III. These findings strengthen the argument that skin biopsy and CCM may be useful biomarkers in PD. Of course our study was not designed to assess the utility of these measures as biomarkers and is limited by the cross sectional design and the assessment of UPDRS-III in the “ON” state, potentially affecting the ability to interpret the motor severity data. Furthermore, skin tissue co-immunostaining with vasoactive intestinal peptide (VIP) or tyrosine hydroxylase would have been required to detect sympathetic adrenergic fibers and differentiate autonomic from sensory nerve involvement. These limitations, however, did not affect our primary objective, which was to assess corneal nerve pathology in PD compared to healthy controls and this was demonstrated with significant effect size.

Corneal confocal microscopy, a rapid non-invasive imaging technique, allows *in vivo* visualization and quantification of corneal nerves. This enables longitudinal assessment of small nerve fiber pathology, which is not readily achieved using skin biopsies in PD. Further longitudinal studies using UPDRS-III “OFF” scores are needed to establish its relationship to motor deficits. Whether CCM can indeed fulfil the criteria of a non-invasive biomarker to detect sub-clinical disease, predict progression and the effect of treatment in PD, remains to be answered.

## Authors roles

All authors have approved the final manuscript.

**Lewis Kass-Iliyya:** Design of the study; acquisition of data; analysis and interpretation of data; drafting the article and revising it critically for important intellectual content; final approval of the version to be submitted. **Saad Javed:** Analysis of data, revising the article critically for important intellectual content; final approval of the version to be submitted. **David Gosal:** Acquisition of data; revising the article critically for important intellectual content; final approval of the version to be submitted. **Christopher Kobylecki:** Conception and design of the study; acquisition of data; revising the article critically for important intellectual content; final approval of the version to be submitted. **Andrew Marshall:** Acquisition of data; analysis of data; final approval of the version to be submitted. **Ioannis N. Petropoulos:** Acquisition of data, revising the article critically for important intellectual content; final approval of the version to be submitted. **Mitra Tavakoli:** Acquisition of data, revising the article critically for important intellectual content; final approval of the version to be submitted. **Maryam Ferdousi:** Acquisition of data, revising the article critically for important intellectual content; final approval of the version to be submitted. **Kallol Ray Chaudhuri:** Final approval of the version to be submitted. **Maria Jeziorska:** Analysis of data; revising the article critically for important intellectual content; final approval of the version to be submitted. **Rayaz A. Malik:** Conception and design of the study; revising the article critically for important intellectual content; final approval of the version to be submitted. **Monty A. Silverdale:** Conception and design of the study; acquisition of data; analysis and interpretation of data; revising the article critically for important intellectual content; final approval of the version to be submitted.

## Figures and Tables

**Table 1:** Demographics, clinical characteristics and quantitative measures of neuropathy in patients with Parkinson's disease (PD) and controls.

	PD patients (n = 27)	Controls (n = 26)	*P* Value
Gender	10 females, 17 males	11 females, 15 males	0.695
Age (years)	63.0 ± 1.5 (49–77)	60.1 ± 1.3 (51–73)	0.164
Disease duration (years)	6.6 ± 0.9 (0.7–21)	–	–
UPDRS-III	26.6 ± 2.3 (10–53)	–	–
Hoehn and Yahr Stage.	I = 10, II = 13, III = 4	–	–
Cumulative Levodopa dose (g)	750.7 ± 165.8 (0–4145.6)	–	–
SCOPA-AUT	15.22 ± 1.3 (4–30)	–	–
NMSS	59.9 ± 5.2 (2–117)	–	–
King's PD pain scale	21.0 ± 3.0 (0–53)	–	–
SFMPQ	21.0 ± 2.5 (0–51)	–	–
NDS	3 ± 0.5 (0–9)	0.8 ± 1.1 (0–4)	0.001
DB-HRV (beats/min)	15.6 ± 1.3 (6–28)	20.9 ± 2.2 (7–40)	0.023
Supine systolic BP (mm Hg)	128.5 ± 3.7 (106–188)	137.0 ± 3.3 (114–180)	0.089
Supine diastolic BP (mm Hg)	75.3 ± 1.7 (60–90)	76.4 ± 2.1 (56–97)	0.678
Supine heart rate (beats/min)	71.5 ± 2.6 (53–97)	66.0 ± 2.1 (49–89)	0.104
Standing systolic BP (mm Hg)	129.7 ± 4.4 (107–204)	134.9 ± 4.0 (101–189)	0.384
Standing diastolic BP (mm Hg)	77.4 ± 2.6 (48–98)	78.7 ± 1.9 (57–100)	0.680
Standing heart rate (beats/min)	76 ± 2.8 (59–101)	70.8 ± 1.8 (54–90)	0.130
Vibration perception threshold	16.4 ± 1.9 (4.8–41)	9.1 ± 1.3 (0.7–24.5)	0.001
Cold perception threshold°C	20.8 ± 2 (0–29.8)	27.9 ± 0.4 (22.8–31)	0.002
Heat perception threshold°C	42 ± 0.8 (36.1–48.7)	37.7 ± 0.4 (33.6–41.4)	<0.001
Cold pain threshold°C	4 ± 1.5 (0–23.8)	12.7 ± 1.7 (0–23.9)	0.001
Heat pain threshold°C	47.7 ± 0.6 (39–50)	45.5 ± 0.6 (38–50)	0.004

Unless otherwise stated data are shown as mean ± SEM (range). UPDRS-III: Unified Parkinson's Disease Rating Scale, SCOPA-AUT: Scale for Outcomes in Parkinson's Disease – Autonomic Symptoms, NMSS: Non-Motor Symptoms Scale, SFMPQ: Short Form McGill Pain Questionnaire, NDS: Neuropathy Disability Scale, DB-HRV: Deep Breathing Heart Rate Variability. BP: Blood Pressure. Student *t* test was applied except for vibration perception, cold perception, head pain and cold pain thresholds where Mann Whitney U test was used.

**Table 2:** Correlations between corneal nerve measures, intraepidermal nerve fiber density and various clinical and demographic data of PD patients.

	CNFD	CNBD	CNFL	IENFD
Age	−0.362	0.002	−0.104	−0.097
Disease duration	−0.284	−0.181	−0.210	−0.416*
Cumulative l-dopa dose	−0.301	−0.243	−0.170	−0.476*
UPDRS-III	−0.337	−0.445*	−0.416*	−0.441*
SCOPA-AUT	−0.373	−0.439*	−0.405*	−0.267
DB-HRV	0.537**	0.681***	0.708***	0.174
NMSS	−0.042	−0.204	−0.047	−0.353
King's PD pain scale	−0.360	−0.232	−0.243	−0.281
SFMPQ	−0.053	−0.249	−0.117	−0.071
NDS	−0.522**	−0.328	−0.360	−0.451*
Vibration threshold	−0.133	−0.278	−0.293	−0.354
Cold threshold	−0.090	−0.242	−0.144	0.227
Heat threshold	−0.385	−0.240	−0.282	−0.399
Cold pain threshold	0.025	−0.303	−0.265	−0.369
Heat pain threshold	−0.336	−0.169	−0.247	−0.094

*P ≤ 0.05, **P ≤ 0.01, ***P ≤ 0.001. Pearson's correlation was applied in all measures except for Levodopa dose, vibration threshold, cold and heat pain thresholds where the non-parametric Spearman's correlation was applied. CNFD: Corneal Nerve Fiber Density, CNBD: Corneal Nerve Branch Density, CNFL: Corneal Nerve Fiber Length, IENFD: Intraepidermal Nerve Fiber Density, UPDRS-III: Unified Parkinson's Disease Rating Scale, SCOPA-AUT: Scale for Outcomes in Parkinson's Disease – Autonomic Symptoms, DB-HRV: Deep Breathing Heart Rate Variability, NMSS: Non-Motor Symptoms Scale, SFMPQ: Short Form McGill Pain Questionnaire, NDS: Neuropathy Disability Scale.

## References

[1] Dabby R., Djaldetti R., Shahmurov M., Treves T.A., Gabai B., Melamed E. (2006). Skin biopsy for assessment of autonomic denervation in Parkinson's disease. J. Neural Transm..

[2] Donadio V., Incensi A., Leta V., Giannoccaro M.P., Scaglione C., Martinelli P. (2014). Skin nerve alpha-synuclein deposits: a biomarker for idiopathic Parkinson disease. Neurology.

[3] Fujishiro H., Frigerio R., Burnett M., Klos K.J., Josephs K.A., Delledonne A. (2008). Cardiac sympathetic denervation correlates with clinical and pathologic stages of Parkinson's disease. Mov. Disord..

[4] Lebouvier T., Neunlist M., Bruley des Varannes S., Coron E., Drouard A., N'Guyen J.M. (2010). Colonic biopsies to assess the neuropathology of Parkinson's disease and its relationship with symptoms. PLoS One.

[5] Wang N., Gibbons C.H., Lafo J., Freeman R. (2013). alpha-Synuclein in cutaneous autonomic nerves. Neurology.

[6] Ceravolo R., Cossu G., Bandettini di Poggio M., Santoro L., Barone P., Zibetti M. (2013). Neuropathy and levodopa in Parkinson's disease: evidence from a multicenter study. Mov. Disord..

[7] Doppler K., Ebert S., Uceyler N., Trenkwalder C., Ebentheuer J., Volkmann J. (2014). Cutaneous neuropathy in Parkinson's disease: a window into brain pathology. Acta Neuropathol..

[8] Nolano M., Provitera V., Estraneo A., Selim M.M., Caporaso G., Stancanelli A. (2008). Sensory deficit in Parkinson's disease: evidence of a cutaneous denervation. Brain.

[9] Rajabally Y.A., Martey J. (2013). Levodopa, vitamins, ageing and the neuropathy of Parkinson's disease. J. Neurol..

[10] Toth C., Brown M.S., Furtado S., Suchowersky O., Zochodne D. (2008). Neuropathy as a potential complication of levodopa use in Parkinson's disease. Mov. Disord..

[11] author reply 8–9.

[12] Muller L.J., Marfurt C.F., Kruse F., Tervo T.M. (2003). Corneal nerves: structure, contents and function. Exp. Eye Res..

[13] Quattrini C., Tavakoli M., Jeziorska M., Kallinikos P., Tesfaye S., Finnigan J. (2007). Surrogate markers of small fiber damage in human diabetic neuropathy. Diabetes.

[14] Tavakoli M., Begum P., McLaughlin J., Malik R.A. (2015). Corneal confocal microscopy for the diagnosis of diabetic autonomic neuropathy. Muscle Nerve.

[15] Asghar O., Petropoulos I.N., Alam U., Jones W., Jeziorska M., Marshall A. (2014). Corneal confocal microscopy detects neuropathy in subjects with impaired glucose tolerance. Diabetes Care.

[16] Ziegler D., Papanas N., Zhivov A., Allgeier S., Winter K., Ziegler I. (2014). Early detection of nerve fiber loss by corneal confocal microscopy and skin biopsy in recently diagnosed type 2 diabetes. Diabetes.

[17] Tavakoli M., Mitu-Pretorian M., Petropoulos I.N., Fadavi H., Asghar O., Alam U. (2013). Corneal confocal microscopy detects early nerve regeneration in diabetic neuropathy after simultaneous pancreas and kidney transplantation. Diabetes.

[18] Tavakoli M., Marshall A., Banka S., Petropoulos I.N., Fadavi H., Kingston H. (2012). Corneal confocal microscopy detects small-fiber neuropathy in Charcot–Marie–Tooth disease type 1A patients. Muscle Nerve.

[19] Tavakoli M., Marshall A., Pitceathly R., Fadavi H., Gow D., Roberts M.E. (2010). Corneal confocal microscopy: a novel means to detect nerve fibre damage in idiopathic small fibre neuropathy. Exp. Neurol..

[20] Tavakoli M., Marshall A., Thompson L., Kenny M., Waldek S., Efron N. (2009). Corneal confocal microscopy: a novel noninvasive means to diagnose neuropathy in patients with Fabry disease. Muscle Nerve.

[21] Reddy V.C., Patel S.V., Hodge D.O., Leavitt J.A. (2013). Corneal sensitivity, blink rate, and corneal nerve density in progressive supranuclear palsy and Parkinson disease. Cornea.

[22] Chaudhuri K.R., Martinez-Martin P., Brown R.G., Sethi K., Stocchi F., Odin P. (2007). The metric properties of a novel non-motor symptoms scale for Parkinson's disease: results from an international pilot study. Mov. Disord..

[23] Chaudhuri K.R., Rizos A., Trenkwalder C., Rascol O., Pal S., Martino D. (2015). King's Parkinson's disease pain scale, the first scale for pain in PD: an international validation. Mov. Disord..

[24] Visser M., Marinus J., Stiggelbout A.M., Van Hilten J.J. (2004). Assessment of autonomic dysfunction in Parkinson's disease: the SCOPA-AUT. Mov. Disord..

[25] Young M.J., Boulton A.J., MacLeod A.F., Williams D.R., Sonksen P.H. (1993). A multicentre study of the prevalence of diabetic peripheral neuropathy in the United Kingdom hospital clinic population. Diabetologia.

[26] Lauria G., Hsieh S.T., Johansson O., Kennedy W.R., Leger J.M., Mellgren S.I. (2010). European Federation of Neurological Societies/Peripheral Nerve Society Guideline on the use of skin biopsy in the diagnosis of small fiber neuropathy. Report of a joint task force of the European Federation of Neurological Societies and the Peripheral Nerve Society. Eur. J. Neurol..

[27] Schneider C., Bucher F., Cursiefen C., Fink G.R., Heindl L.M., Lehmann H.C. (2014). Corneal confocal microscopy detects small fiber damage in chronic inflammatory demyelinating polyneuropathy (CIDP). J. Peripher Nerv. Syst..

[28] Azmi S., Ferdousi M., Petropoulos I.N., Ponirakis G., Fadavi H., Tavakoli M. (2015). Corneal confocal microscopy shows an improvement in small-fiber neuropathy in subjects with type 1 diabetes on continuous subcutaneous insulin infusion compared with multiple daily injection. Diabetes Care.

[29] Petropoulos I.N., Alam U., Fadavi H., Asghar O., Green P., Ponirakis G. (2013). Corneal nerve loss detected with corneal confocal microscopy is symmetrical and related to the severity of diabetic polyneuropathy. Diabetes Care.

[30] Beiske A.G., Loge J.H., Ronningen A., Svensson E. (2009). Pain in Parkinson's disease: prevalence and characteristics. Pain.

